# A Novel 3,9-(1,2,3-Trioxocine)-Type Steroid of *Rauia nodosa* (Rutaceae)

**DOI:** 10.3390/molecules190914637

**Published:** 2014-09-16

**Authors:** Michelle Rodrigues e Rocha, Jucimar Jorgeane de Souza, Lucas Tricarico Barcellos, Carlos Mauricio R. Sant’Anna, Raimundo Braz-Filho, Ivo José Curcino Vieira

**Affiliations:** 1 Laboratório de Ciências Químicas, Universidade Estadual do Norte Fluminense Darcy Ribeiro, Campos dos Goytacazes 28013-602, Brazil; 2 Departamento de Química, Instituto de Ciência Exatas, Universidade Federal Rural do Rio de Janeiro, Seropédica 23890-000, Brazil

**Keywords:** Rutaceae, *Rauia nodosa*, steroids, alkaloids, coumarins

## Abstract

A new natural product, a 3,9-(1,2,3-trioxocine)-type steroid, named rauianodoxy (**6**), was isolated from *Rauia nodosa*, together with five steroids: sistostenone (**1**), stigmastenone (**2**), sitosterol (**3**), stigmasterol (**4**) and ergosterol peroxide (**5**), one coumarin, *O*-geranylosthenol (**7**), and three alkaloids, *N*-methylflindersine (**8**), zantobungeanine (**9**) and veprissine (**10**). Compounds **5**–**8** were isolated for the first time in the genus *Rauia*. These compounds were characterized on the basis of their spectral data, mainly one and two-dimensional NMR, and mass spectra, also involving comparison with the literature data. Theoretical studies at the DFT level reveal structural parameters for the 1,2,3-trioxole bridge compatible with known structures containing a similar group.

## 1. Introduction

The Rutaceae family is represented by 155 genera and 1600 species distributed in the tropical and temperate regions of the World. This family is commonly found in Tropical America, South Africa, Asia and Australia [1]. The Rutaceae family is characterized by an abundance of anthranilic acid derived alkaloids, coumarins, limonoids and flavonoids, with different types of biological activities [2]. The biological importance of compounds from the Rutaceae family can be highlighted by the *Pilocarpus* genus that synthesizes large concentrations of the alkaloid pilocarpine used to treat glaucoma [3].

The *Rauia* genus, taxonomically found in the Rutaceae family, Rutoideae subfamily, Galipeae tribe (formerly Cusparieae) and Galipeinae subtribe (before Cuspariinae) [4] has been little studied. This genus includes five species: *R. spicata*, *R. subtruncata*, *R. nodosa*, *R. prancei* and *R. resinosa*. The last three species can be found in the North, Northeast and Southeast Brazil. The phytochemical study of *R. resinosa* revealed the presence of alkaloids and coumarins [5].

In the present paper, we describe the isolation and characterization of a novel 3,9-(1,2,3-trioxocine)-type steroid named rauianodoxy (**6**), together with nine known compounds: five steroids, a mixture of sistostenone (**1**) and stigmastenone (**2**), a mixture of sitosterol (**3**) and stigmasterol (**4**) and ergosterol peroxide (**5**), one coumarin, *O*-geranylosthenol (**7**), and three alkaloids, a mixture of *N*-methylflindersine (**8**) and zantobungeanine (**9**), and veprissine (**10**). All these natural products were isolated for the first time in this species and the 3,9-(1,2,3-trioxocine)-type steroid named rauianodoxy (**6**) is mentioned for the first time on record. The compounds **5**–**8** were isolated for the first time in the *Rauia* genus. The structures were established on the basis of spectral data, mainly ^1^H and ^13^C (1D and 2D) NMR spectra, mass spectrometry and by comparison with literature data. Complementary results describing structural parameters of the novel steroid were obtained from calculations at the DFT level.

## 2. Results and Discussion

**Figure 1:**
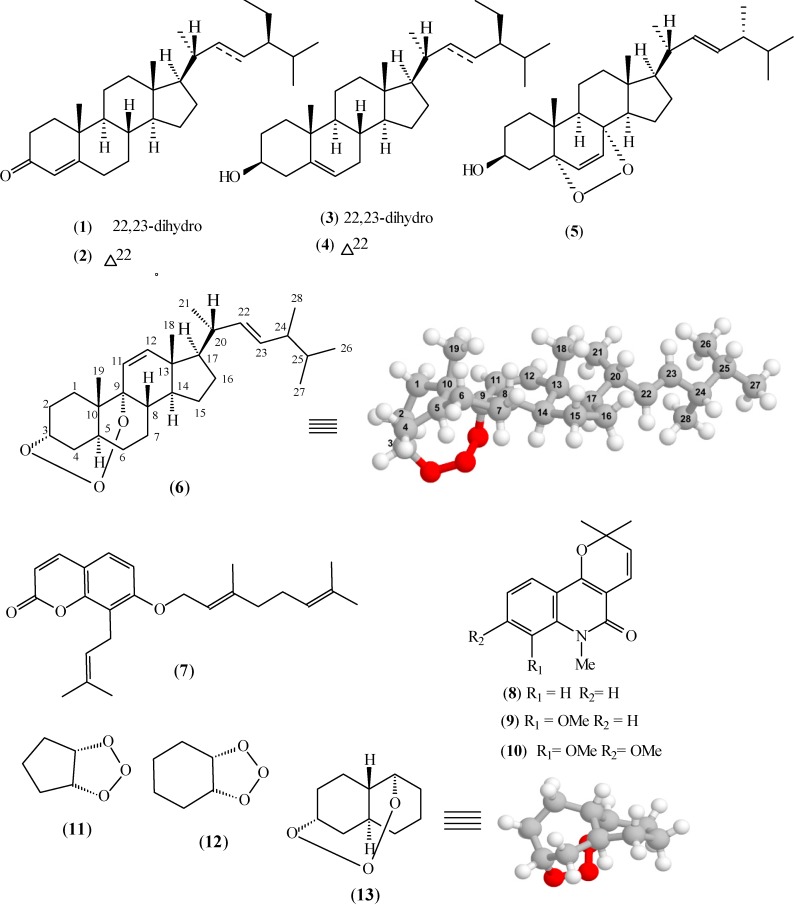
Compounds isolated from the stems of *Rauia nodosa* (**1**–**10**) and compounds **11**–**13**.

The hexane extract of stems of *R. nodosa* was subjected to a chromatographic method to obtain the new 3,9-(1,2,3-trioxocine)-type steroid, named rauianodoxy (**6**), together with the five known steroids, sistostenone (**1**) and stigmastenone (**2**) in a mixture [6,7], sitosterol (**3**) and stigmasterol (**4**) in a mixture [7,8], and ergosterol peroxide (**5**) [9], one coumarin, *O*-geranylosthenol (**7**) [10], and three alkaloids, *N*-methylflindersine (**8**) [11,12] and zantobungeanine (**9**) in a mixture [13], and veprissine (**10**) [13]. The structures were identified on the basis of ^1^H- and ^13^C-NMR spectral data, including 2D NMR experiments [14,15] which were also used to complete and clearly define the ^1^H and ^13^C chemical shift assignments of steroid **6**, the new natural product (see Figure 1).

**Scheme 1:**
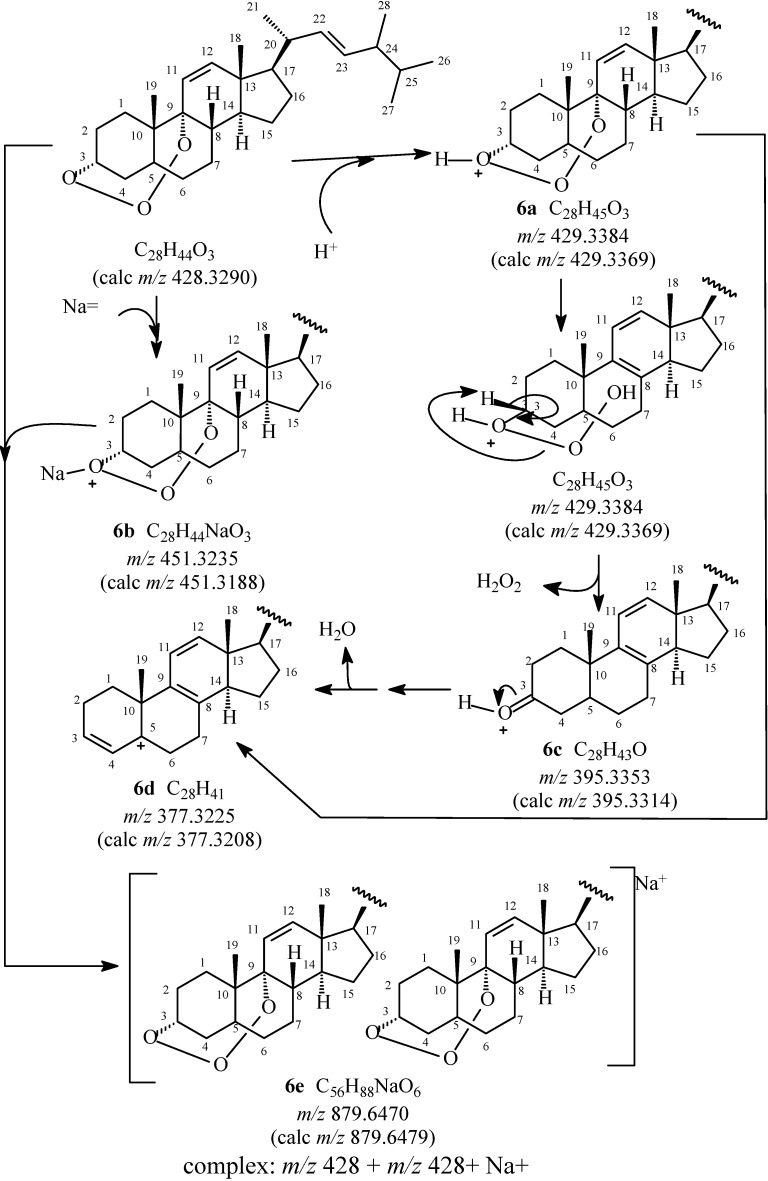
Proposed fragmentation mechanisms to justify peaks (positive modes, only peaks classified as principal) obtained in a methanol/water solution of compound **6**.

Rauianodoxy (**6**) 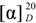 = −10.2, (MeOH, *c* 1.0), was obtained as yellow oil. Analysis of the DEPTQ-^13^C-NMR spectrum (Table 1), involving the corroboration of ^1^H and ^13^C-NMR spectra, allowed to recognize the presence of 28 signals corresponding to three non-hydrogenated [(C)_3_: all sp^3^ (including one oxygenated at δ_C_ 82.2], twelve methine [(CH) × 12: eight sp^3^ (including one oxygenated at δ_C_ 66.4) and four sp^2^ olefinic at δ_C_ 135.5, 130.8, 135.2 and 132.2], seven methylene [(CH_2_) × 7, all sp^3^] and six methyl [(CH_3_) × 6] carbon atoms, allowing to deduce the presence of C_28_H_44_O_2_, HRESIMS (positive mode) revealed [M+H]^+^ at *m/z* 429.3384 (**6a**, C_28_H_45_O_3_, required *m/z* 429.3369) and [M+Na]^+^ at *m/z* 451.3235 (**6b**, C_28_H_44_NaO_3_, required *m/z* 451.3188) compatible with the molecular formulae C_28_H_44_O_3_ (Scheme 1). The presence of double bonds located at carbon atoms CH-11 [δ_C_ 135.5/δ_H_ 6.27 (*d*, *J* = 8.5 Hz)]/CH-12 [δ_C_ 130.8/δ_H_ 6.53 (*d*, *J* = 8.5 Hz)] and CH-22 [δ_C_ 135.2/δ_H_ 5.17 (*dd*, *J* = 8.5, coupling of H-22 and H-20, and 15.2, coupling of H-22 and H-23, Hz)]/CH-23 [δ_C_ 132.2/δ_H_ 5.23 (*dd*, *J* = 8.2, coupling of H-23 and H-24, and 15.2 Hz, coupling of H-23 and H-22)] in an stigmasterol skeleton (e.g., **4**, stigmasterol, [4,13]). In fact, heteronuclear long-range coupling of these carbon atoms [CH-12 (δ_C_ 130.8) with 3H-18 (δ_H_ 0.84), CH-22 (δ_C_ 135.2) with 3H-21 (δ_H_ 1.02) and CH-23 (δ_C_ 132.2) with 3H-28 (δ_H_ 0.92)] confirming the presence of two double bonds in the steroidal-skeleton **6**, as shown in Table 1.

The ^13^C-NMR spectrum of **6** revealed only two oxygenated carbon atoms at δ_C_ 66.4 [CH-3, which revealed heteronuclear interaction with 2H-4 (δ_H_ 1.97 and 1.25) in the HMBC spectrum] and 82.2 [C-9, which revealed heteronuclear interaction with H-11 (δ_H_ 6.27), H-12 (δ_H_ 6.53) and 3H-19 (δ_H_ 0.91) in the HMBC spectrum] and the molecular formulae C_28_H_44_O_3_ (two double bonds and pentacyclic skeleton) induced us inevitably to postulate the presence of the O-O-O (O_3_) group and to classify the new compound as a 1,2,3-trioxocine-type steroid **6** (see Table 1). Thus, the observed signals in the one-dimensional ^13^C-NMR spectrum and corroboration by the observed signals in the two-dimensional HMBC spectra obtained for the compound **6** allowed us to postulate the presence of the 1,2,3-trioxocine group involving the carbon atoms CH-3 and C-9 and to confirm the location of one double bond between the carbon atoms CH-11 and CH-12 (**6**, Table 1). Additional heteronuclear long-range couplings are summarized in Table 1.

**Table 1:** ^1^H- (500 MHz) and ^13^C- (125 MHz) NMR of rauianodoxy (**6**), including results obtained by heteronuclear 2D shift-correlated HSQC and HMBC, in CDCl_3_ as solvent and TMS used as internal reference. Chemical shifts (δ, ppm) and coupling constants (*J*, Hz, in parenthesis).

	HSQC		HMBC	
	δ_C_	δ_H_	^2^*J*_HC_	^3^*J*_HC_
**C**				
9	82.2	-	H-11	H-12; 3H-19
10	38.2	-	3H-19	H-11
13	44.4	-	3H-18	
**CH**				
3	66.4	4.02 (*br s*)	H-4	
5	51.1	1.50–1.55 (*m*)		3H-19
8	38.3	1.28–1.30 (*m*)		
11	135.5	6.27 (*d*, 8.5)		
12	130.8	6.53 (*d*, 8.5)		3H-18
14	51.7	1.58–1.59 (*m*)		3H-18
17	56.1	1.24–1.25 (*m*)		3H-18; 3H-21
20	39.7	2.01–2.07 (*m*)	3H-21	H-23
22	135.2	5.17 (*dd*, 15.2, 8.5)		3H-21
23	132.2	5.23 (*dd*, 15.2, 8.2)	H-24	3H-28
24	42.8	1.87–1.89 (*m*)	3H-28	H-22; 3H-26; 3H-27
25	33.1	1.48–1.5 (*m*)	3H-26; 3H-27	3H-28
**CH**_2_				
1	36.9	2.15–2.17 (*m*); 1.90–2.06 (*m*)		3H-19
2	34.7	1.93–1.95 (*m*); 1.65–1.68 (*m*)		
4	38.3	1.97–2.0 (*m*); 1.25–1.31(*m*)		
6	20.7	1.63–1.65 (*m*); 1.43–1.44 (*m*)		
7	30.1	1.85–1.87 (*m*); 1.60–1.61 (*m*)		
15	23.4	1.53–1.55 (*m*); 1.23–1.25 (*m*)		
16	28.7	1.71–1.75 (*m*); 1.30–1.35 (*m*)		
**CH**_3_				
18	12.9	0.84 (*s*)		
19	18.2	0.91 (*s*)		
21	20.8	1.02 (*d*, 6.5)		
26	20.0	0.85 (*d*, 7.0)		3H-27
27	19.7	0.84 (*d*, 6.9)		3H-26
28	17.6	0.92 (*d*, 7.4)		

* Number of hydrogens bound to carbon atoms deduced by DEPTQ-^13^C-NMR spectrum. Chemical shifts and coupling constants (*J*) obtained of 1D ^1^H-NMR spectrum. Superimposed ^1^H signals are described without multiplicity and chemical shifts deduced by HMQC, HMBC and ^1^H-^1^H-COSY spectra.

**Figure 2:**
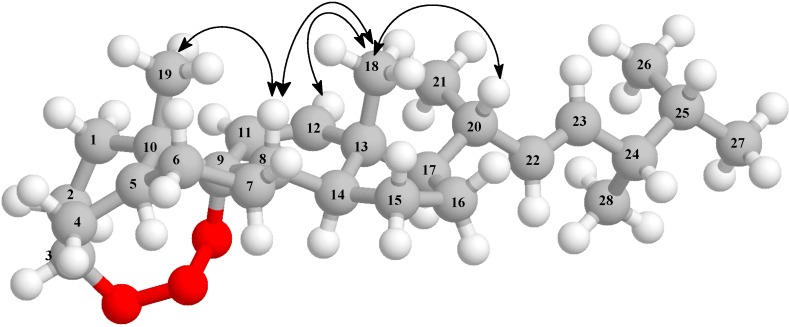
Select (NOE) spatial correlations and relative stereochemistry for 3,9-(1,2,3-trioxocine)-type steroid rauianodoxy (**6**). Arrows denote NOE spatial correlations principals.

The relative stereochemistry of rauianodoxy (**6**) was determined from the coupling constants of relevant hydrogen atoms and from the observed ^1^H-^1^H-NOESY, which showed cross-peaks assigned to dipolar interaction (spatial proximity, as shown in Figure 2). Thus, the observed hydrogen H-8 spatial interactions with both methyl groups 3H-19 and 3H-18 indicated that these hydrogen atoms are in β-orientations (axial-axial); the relative configuration *S* assigned to the CH-20 carbon atom was based on the special interaction between 3H-18 and H-20, as shown in Figure 2.

The results of the extensive application of 1D and 2D NMR spectral techniques were also used to confirm the structure and to establish the complete ^1^H and ^13^C chemical shift assignments of **6** (Table 1).

The HRESIMS obtained for the compound **6**, essential to postulate the presence of one additional oxygen atom and to define the molecular formulae C_28_H_44_O_3_ by [M+H]^+^ at *m/z* 429.3384 (C_28_H_45_O_3_), [M+Na]^+^ at *m/z* 451.3235 (C_28_H_44_NaO_3_), and the adduct [2M+Na]^+^ at *m/z* 879.6479 (C_56_H_88_NaO_6_, required *m/z* 879.6470), was also useful in confirming the new steroidal 3,9-(1,2,3-trioxocine)-type structure mainly due to fragment with *m/z* 395.3353 (C_28_H_43_O, required *m/z* 395.3314), formed by elimination of a molecule of hydrogen peroxide (H_2_O_2_). This fragment was the precursor for the formation of fragment with *m/z* 377.3225 (C_28_H_31_, required *m/z* 377.3208), a result of the loss of a water molecule (H_2_O) (Scheme 1). Alternatively, this fragment of *m/z* 377.3225 may also be produced from [M+H]^+^ by simultaneous eliminations of H_2_O_2_ and H_2_O.

A few natural ozonides (structures containing the 1,2,3-trioxocine group) as precursors in ozonolysis of olefins, have been discovered in higher plant species [16,17,18]. However, plant ozonides could be formed from cuticle olefins with ozone from the air, or alternatively inside cells, as ozone may enter the mesophyll of the plants [19,20] causing necrosis of the leaves.

**Figure 3:**
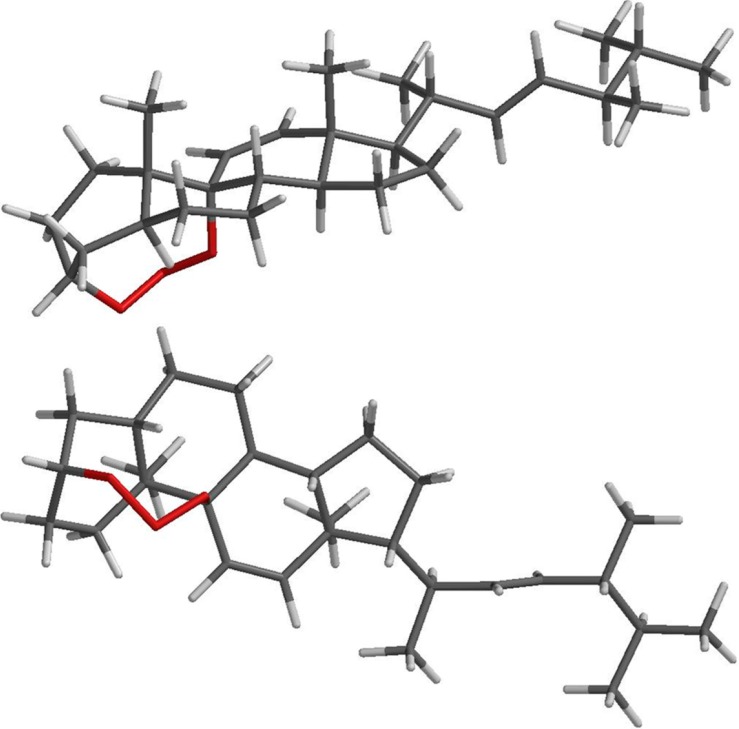
Perpendicular views of the 3D representation of LSDA/pBP86/DN* optimized structure of the 3,9-(1,2,3-trioxocine)-type steroid, compound **6**.

Compound **6** was mainly characterized by one and two-dimensional NMR and mass spectra, but theoretical studies at the DFT level have also been used. A model was performed to access structural details of the suggested structure and to verify the stability of the 1,2,3-trioxocine bridge connecting two atoms, C3 and C9, spatially not very close to each other in compound **6**. The modeling results revealed that the proposed bridge is in fact stable, with bonding distances comparable to those of known compounds (models) containing similar bridges. In the optimized LSDA/pBP86/DN* compound **6** structure, the 1,2,3-trioxocine bridge is part of two adjacent eight-membered rings which adopt two different conformations: a distorted chair-like conformation and a distorted boat-like conformation (Figure 3). The C(3)-O(1) distance was 1.470 Å and C(9)-O(3) distance was 1.520 Å.

Structures containing the proposed 1,2,3-trioxocine bridge are scarce, so it is difficult to obtain structural data of known compounds to compare with the proposed structure; the majority of 1,2,3-trioxocine bridge-containing species reported in the literature came from reactions between O_3_ and small alkenes and cycloalkenes, which are quite unstable. Therefore, in order to verify the adequacy of the modeled structure, we modeled by the same procedure the structures of the known products of the reactions of cyclopentene and cyclohexene with O_3_ [21], both containing trioxole groups. The C-O distances in the compound that results from the reaction between O_3_ and cyclopentene (compound **11** in Figure 1), after geometry optimization, were 1.439 and 1.440 Å. In the case of the cyclohexene derivative (compound **12** in Figure 1), these distances were 1.425 and 1.442 Å. The longer bond distances obtained for compound **6** could be attributed to the long distance between the atoms to which O(1) and O(3) atoms are attached, C(3) and C(9), which are part of the rigid steroid ring system, and also to 1,3-diaxial steric interactions between the O(1) and O(3) atoms with H atoms. Accordingly, in a simplified model of compound **6**, containing only rings A and B (compound **13** in Figure 1), modeled by the same procedure, the C(3)-O(1) and C(9)-O(3) distances were also longer, 1.464 Å was 1.475 Å, respectively.

The 1,2,3-trioxocine bridge is not symmetrical in the steroid derivative, with the O(1)-O(2) distance is equal to 1.507 Å and the O(2)-O(3) distance is equal to 1.471 Å. The corresponding bond distances were almost identical in the model compound **11**, 1.474 and 1.470 Å, but were clearly different in the case of compounds **12** and **13**, 1.579 and 1.423 Å, 1.538 and 1.444 Å, respectively. In compound **6**, the O(1)-O(2)-O(3) angle is 112.57°, a value compatible with a sp^3^ character for the oxygen atoms. Thus, the structure of the new 3,9-(1,2,3-trioxocine)-type steroid isolated from *Rauia nodosa* was here established for the first time as a novel natural product and named rauianodoxy (**6**).

## 3. Experimental Section

### 3.1. General Experimental Procedures

ESI-MS (high resolution) mass spectra were obtained by using a ESI-TOF-MS Shimadzu mass spectrometer (SHIMADZU, Universidade Estadual do Norte Fluminense, Campos dos Goytacazes, Brazil), using the positive ion mode of analysis. Chromatographic purifications were carried out by using silica gel 60 (0.063–0.200 mm). ^1^H- and ^13^C-NMR spectra were measured on a Brüker Ultrashield 500 Plus spectrometer (BRÜKER, Universidade Federal Rural do Rio de Janeiro, Seropédica, Brazil), operating at 500 (^1^H) and 125 (^13^C) MHz. CDCl_3_ was used as solvent with TMS as internal reference. Chemical shifts are given in the δ scale (ppm) and coupling constants (*J*) in Hz. One dimensional (1D) ^1^H- and ^13^C-NMR spectra were acquired under standard conditions by using a direct detection 5 mm ^1^H/^13^C dual probe. Standard pulse sequences were used for two dimensional spectra by using a multinuclear inverse detection 5 mm probe with field gradient.

A theoretical study was implemented to verify the viability of the molecular structure containing the 1,2,3-trioxocine bridge. The Spartan’10 (Wavefunction, Inc., Universidade Federal Rural do Rio de Janeiro, Seropédica, Brazil) program was used in all steps of the theoretical study. The structure was initially submitted to a Monte Carlo conformational search with the MMFF94 molecular mechanics method [22]. This procedure generated a number of conformers and the geometry of the most stable conformer was then fully reoptimized in two steps: first with the semiempirical molecular orbital PM3 method [23], and finally with the pBP86/DN* DFT method. The pBP86, composed of the Becke 1988 exchange functional [24] and the Perdew 86 correlation functional [25], provides a perturbative implementation of the non-local Becke-Perdew model. The DN* basis set is derived from numerical atomic solutions, incorporating polarization functions on heavy (*i.e.*, non-hydrogen) atoms, which adds some flexibility within the basis set.

### 3.2. Plant Material

Stems of *Rauia nodosa* (Rutaceae) were collected in May 2011 at Vale Cia Reserve, Linhares, Espírito Santo, Brazil. The voucher specimen of *R. nodosa* was deposited at Vale Cia herbarium, under the code CRVD-3301.

### 3.3. Extraction and Isolation

Stems of *Rauia nodosa* (Rutaceae), immediately after collection, were dried at room temperature until a constant weight (about one week) and ground in hammer mills. The dried and powdered material (4.8 kg) was extracted with hexane. The obtained solutions (48 L) were distilled under reduced pressure in a rotary evaporator furnishing 4.47 g of crude hexane extract.

The hexane extract was chromatographed over silica gel column with a gradient of hexane/ethyl acetate to afford twenty one fractions. Fraction 9 (835.6 mg) was rechromatographed over a silica gel column with a gradient of hexane/ethyl acetate furnishing six fractions. Fraction 9.2 (62.8 mg) was rechromatographed over a silica gel column with a gradient of hexane/ethyl acetate affording the compounds **1**+**2** (5.1 mg). Fraction 9.5 (260.9 mg) was rechromatographed over a silica gel column with a gradient of hexane/ethyl acetate furnishing seven fractions and **3**+**4** (11.5 mg) and the fraction 9.5.6 (55.6 mg) was rechromatographed over a silica gel column with a gradient of hexane/ethyl acetate yielding compound **7** (29.8 mg).

Fraction 16 (227.2 mg) was rechromatographed over a silica gel column with a gradient of hexane/ethyl acetate affording eleven fractions and the fraction 16.7 (53.2 mg) was rechromatographed over a silica gel column with a gradient of hexane/ethyl acetate yielding compound **5** (5.3 mg). Fraction 10 (292.6 mg) was chromatographed over a silica gel column with a gradient of hexane/ethyl acetate furnishing eight fractions. Fraction 10.8 (94.6 mg) was rechromatographed over a silica gel column with a gradient of hexane/ethyl acetate yielding compound **6** (4.7 mg). Fraction 19 (126.0 mg) was chromatographed over a silica gel column with a gradient of hexane/ethyl acetate affording eight fractions. The fraction 19.5 was chromatographed over a silica gel column with a gradient of hexane/ethyl acetate the provide compounds **8**+**9** (8.8 mg) and **10** (3.7 mg).

*Rauianodoxy* (**6**). 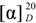 = −10.2, (MeOH, *c* 1.0) and obtained as a yellow oil; HRESIMS (positive mode) *m/z* 429.3384 ([M+H]^+^, calcd. *m/z* 429.3369) and *m/z* 451.3235 ([M+Na]^+^, calcd. *m/z* 451.3188); ^1^H- and ^13^C-NMR data, see Table 1.

*Ergosterol 5,8-peroxide* (**5**). ^1^H-NMR δ_H_ (ppm): 1.97–2.0, 1.71–1.73 (*m*; 2H-1), 1.86–1.90, 1.50–1.52 (*m*; 2H-2), 4.01 (*m*; 1H-3), 2.15–2.17, 1.90–1.97 (*m*; 2H-4), 6.53 (*d*; 8.4; 1H-6), 6.27 (*d*; 8.4; 1H-7), 1.5 (*dd*; 6.95 and 5.99; 1H-9), 1.63–1.67, 1.42–1.45 (*m*; 2H-11), 1.98–2.2, 1.23–1.27 (*m*; 2H-12), 1.58 (*d*; 6.5; 1H-14), 1.3–1.35 (*m*; 2H-15), 1.76–1.80 (*m*; 2H-16), 1.23–1.24 (*m*; 1H-17), 0.84 (*s*; 3H-18), 0.91 (*s*; 3H-19), 2.05–2.15 (*m*; 1H-20), 1.02 (*d*; 6.5; 3H-21), 5.16 (*dd*; 15.3 and 8.2; 1H-22), 5.24 (*dd*; 15.3 and 7.6; 1H-23), 1.87–1.90 (*m*; 1H-24), 1.47–1.49 (*m*; 1H-25), 0.85 (*s*; 3H-26), 0.83 (*s*; 3H-27), 0.93 (*s*; 3H-28); ^13^C-NMR δ_C_ (ppm): 34.7 (CH_2_-1), 30.1 (CH_2_-2), 66.4 (CH-3), 36.9 (CH_2_-4), 81.0 (C-5), 130.8 (CH-6), 135.4 (CH-7), 51.1 (CH-9), 36.6 (C-10), 20.6 (CH_2_-11), 39.8 (CH_2_-12), 44.6 (C-13), 51.7 (CH-14), 20.6 (CH_2_-15), 29.3 (CH_2_-16), 56.2 (CH-17), 12.9 (CH_3_-18), 17.6 (CH_3_-19), 39.8 (CH-20), 20.9 (CH_3_-21), 135.2 (CH-22), 132.3 (CH-23), 42.8 (CH-24), 33.1 (CH-25), 20.0 (CH_3_-26), 19.7 (CH_3_-27), 18.2 (CH_3_-28).

*O-geranylosthenol* (**7**). ^1^H-NMR δ_H_ (ppm): 6.24 (*d*; 9.4; 1H-3), 7.62 (*d*; 9.4; 1H-4), 7.29 (*d*; 8.4; 1H-5), 6.84 (*d*; 8.6; 1H-6), 4.66 (*d*; 6.4; 2H-1'), 5.51 (*t*; 5.54; 1H-2'), 1.73 (*s*; 3H-3'a), 2.11-2.14 (*m*; 2H-4'), 2.14–2.19 (*m*; 2H-5'), 5.10 (*dt*; 5.4; 1H-6'), 1.59 (*s*; 3H-8'), 1.69 (*s*; 3H-9'), 3.57 (*d*; 9.3; 2H-1''), 5.27 (*dt*; 6.1; 1H-2''), 1.90 (*s*; 3H-4'') ,1.67 (*s*; 3H-5''); ^13^C-NMR δ_C_ (ppm): 161.5 (C-2), 112.9 (CH-3), 143.8 (CH-4), 125.0 (CH-5), 108.6 (CH-6), 159.63 (C-7), 118.3 (C-8), 152.9 (C-9), 113.3 (C-10), 65.7 (CH_2_-1'), 119.2 (CH-2'), 141.5 (C-3'), 39.5 (CH_2_-4'), 26.3 (CH_2_-5'), 123.7 (CH-6'), 132.4 (C-7'), 17.7 (CH_3_-8'), 25.8 (CH_3_-9'), 22.1 (CH_2_-1''), 121.3 (CH-2''), 131.9 (C-3''),18.0 (CH_3_-4''), 25.7 (CH_3_-5''), 16.7 (CH_3_-3'a).

*N*-*methylflindersine* (**8**). ^1^H-NMR δ_H_ (ppm): 8.00 (*d*; 7.8; 1H-5), 7.26 (*t*; 7.8; 1H-6), 7.58 (*t*; 7.8; 1H-7), 7.35 (*d*; 8.4; 1H-8), 6.79 (*d*; 9.9; 1H-1'), 5.57 (*d*; 9.9; 1H-2'), 1.57 (*s*; 6H-4'/5'), 3.73 (*s*; CH_3_-N); ^13^C-NMR δ_C_ (ppm): 122.9 (CH-5), 121.7 (CH-6), 130.8 (CH-7), 113.7 (CH-8), 117.5 (CH-1'), 126.3 (CH-2'), 28.0 (CH_3_-4'/5'), 29.2 (CH_3_-N).

*Zantobungeanine* (**9**). ^1^H-NMR δ_H_ (ppm): 7.63 (*d*; 7.8; 1H-5), 7.17 (*t*; 7.8; 1H-6), 7.08 (*t*; 7.8; 1H-7), 6.78 (*d*; 9.9; 1H-1'), 5.56 (*d*; 9.9; 1H-2'), 1.53 (*s*; 6H-4'/5'), 3.93 (*s*; CH_3_O-8), 3.96 (*s*; CH_3_-N); ^13^C-NMR δ_C_ (ppm): 115.6 (CH-5), 122.3 (CH-6), 114.1 (CH-7), 117.8 (CH-1'), 126.4 (CH-2'), 78.5 (C-3'), 28.0 (CH_3_-4'/5'), 56.3 (CH_3_O-8), 35.0 (CH_3_-N).

*Veprissine* (**10**). ^1^H-NMR δ_H_ (ppm): 7.73 (*d*; 8.9; 1H-5), 6.90 (*d*; 8.9; 1H-6), 6.75 (*d*; 9.9; 1H-1'), 5.51 (*d*; 9.9; 1H-2'), 1.52 (*s*; 6H-4'/5'), 3.99 (*s*; CH_3_O-7), 3.80 (*s*; CH_3_O-8), 3.96 (*s*; CH_3_-N); ^13^C-NMR δ_C_ (ppm): 162.6 (C-2), 104.0 (C-3), 155.1 (C-4), 119.1 (CH-5), 107.2 (CH-6), 155.6 (C-7), 136.0 (C-8), 134.6 (C-9), 112.1 (C-10), 118.0 (CH-1'), 125.6 (CH-2'), 78.7 (C-3'), 28.0 (CH_3_-4'/5'), 56.3 (CH_3_O-7), 61.7 (CH_3_O-8), 33.6 (CH_3_-N).

## 4. Conclusions

The hexane extract from the stems of *Rauia nodosa* provided six steroids **1**–**6**, one coumarin **7** and three alkaloids **8**–**10** isolated in a previous phytochemical investigation. The compounds **5**–**8** were isolated for first time in the genus Rauia. There are no published records yet of other steroids possessing 1,2,3-trioxocine group yet. Then, the 3,9-(1,2,3-trioxocine)-type steroid, named rauianodoxy (**6**) was established as novel natural product and consequently described for the first time on in the literature.

## Supplementary Materials

Supplementary materials can be accessed at: http://www.mdpi.com/1420-3049/19/9/14637/s1.

## Supplementary Files

Supplementary File 1
