# Supplementary material for: A Novel 3,9-(1,2,3-Trioxocine)-Type Steroid of Rauia nodosa (Rutaceae)
Source: Molecules. 2014 Sep 16;19(9):14637–48. doi: 10.3390/molecules190914637 (PMC6271486; doi:10.3390/molecules190914637)

Supplementary Materials

Figure S1. Spectra HRESIMS (E+) of compound **6**.

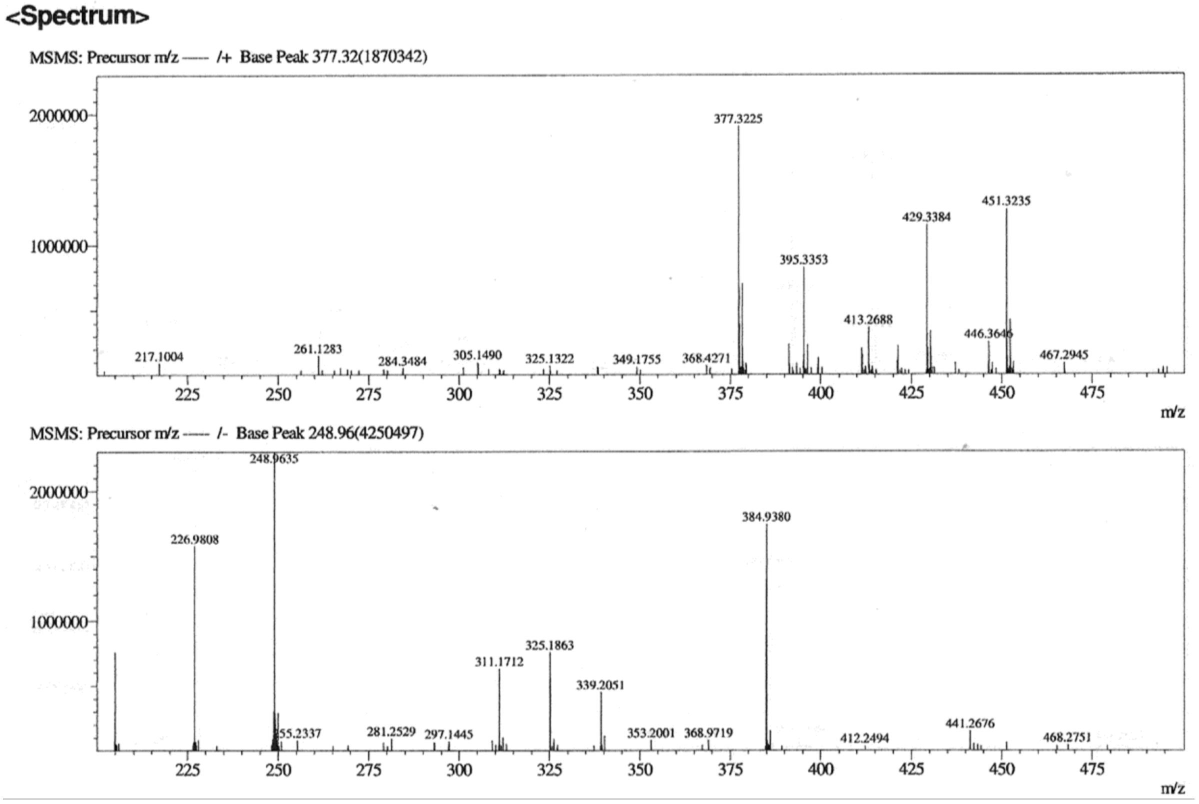

Figure S2. Spectra HRESIMS (E+) of compound **6**.

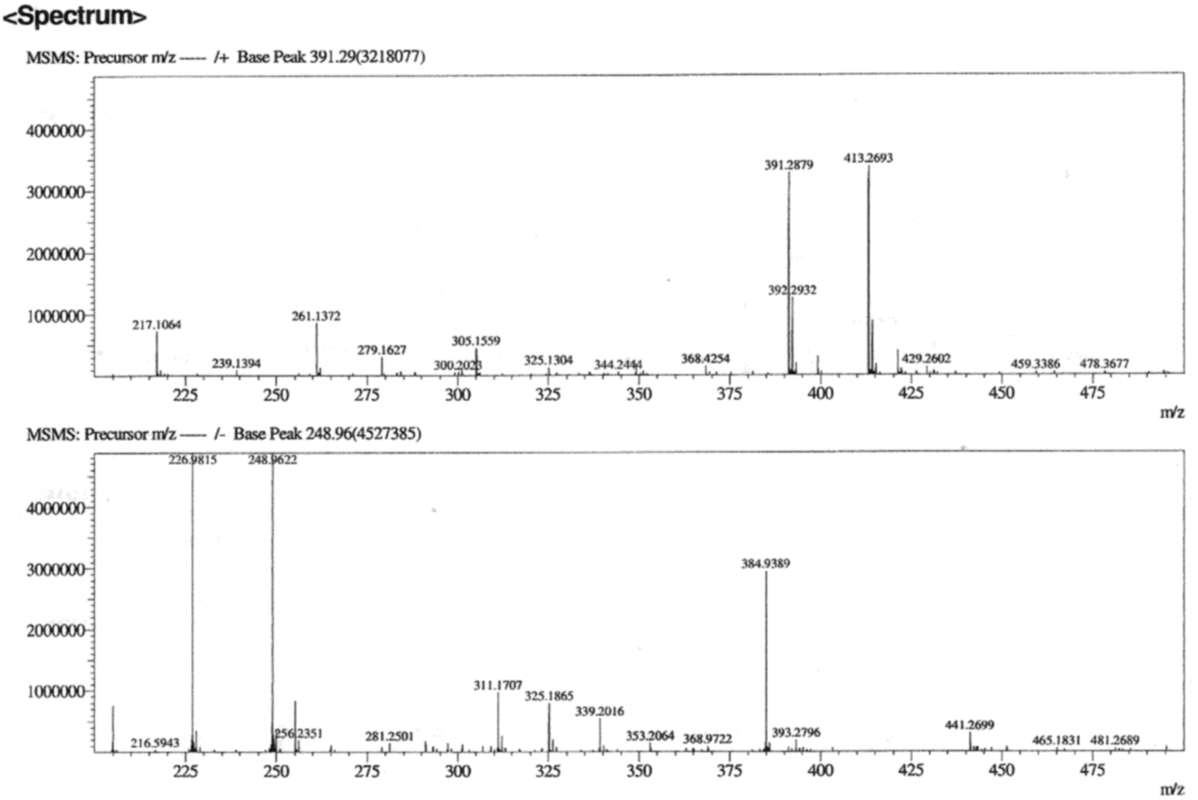

Figure S3. Spectra HRESIMS (E+) of compound **6**.

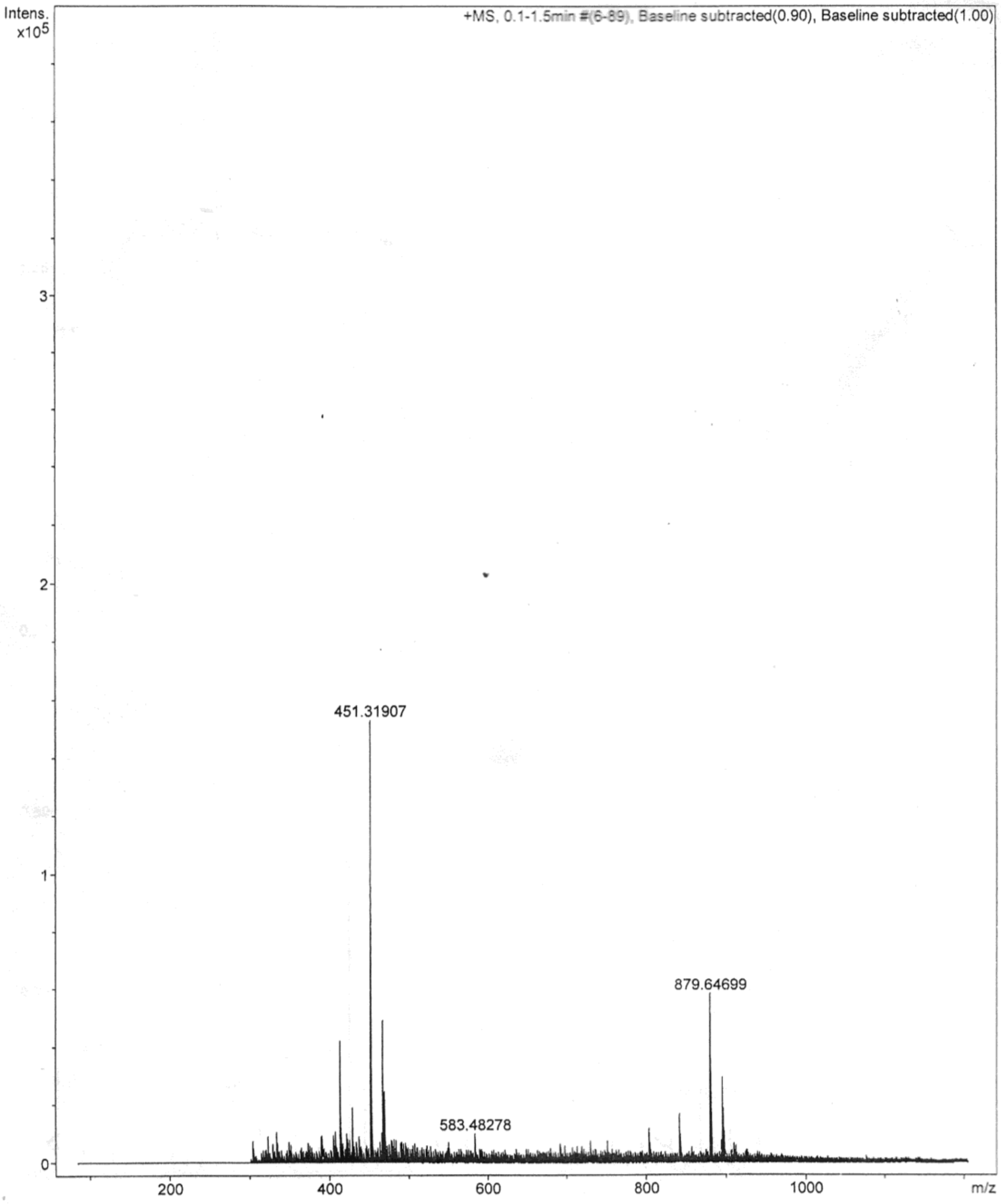

Figure S4. ^{13}C NMR -DEPT Q NMR (125 MHz, $CDCl_3$) spectrum of compound **6**.

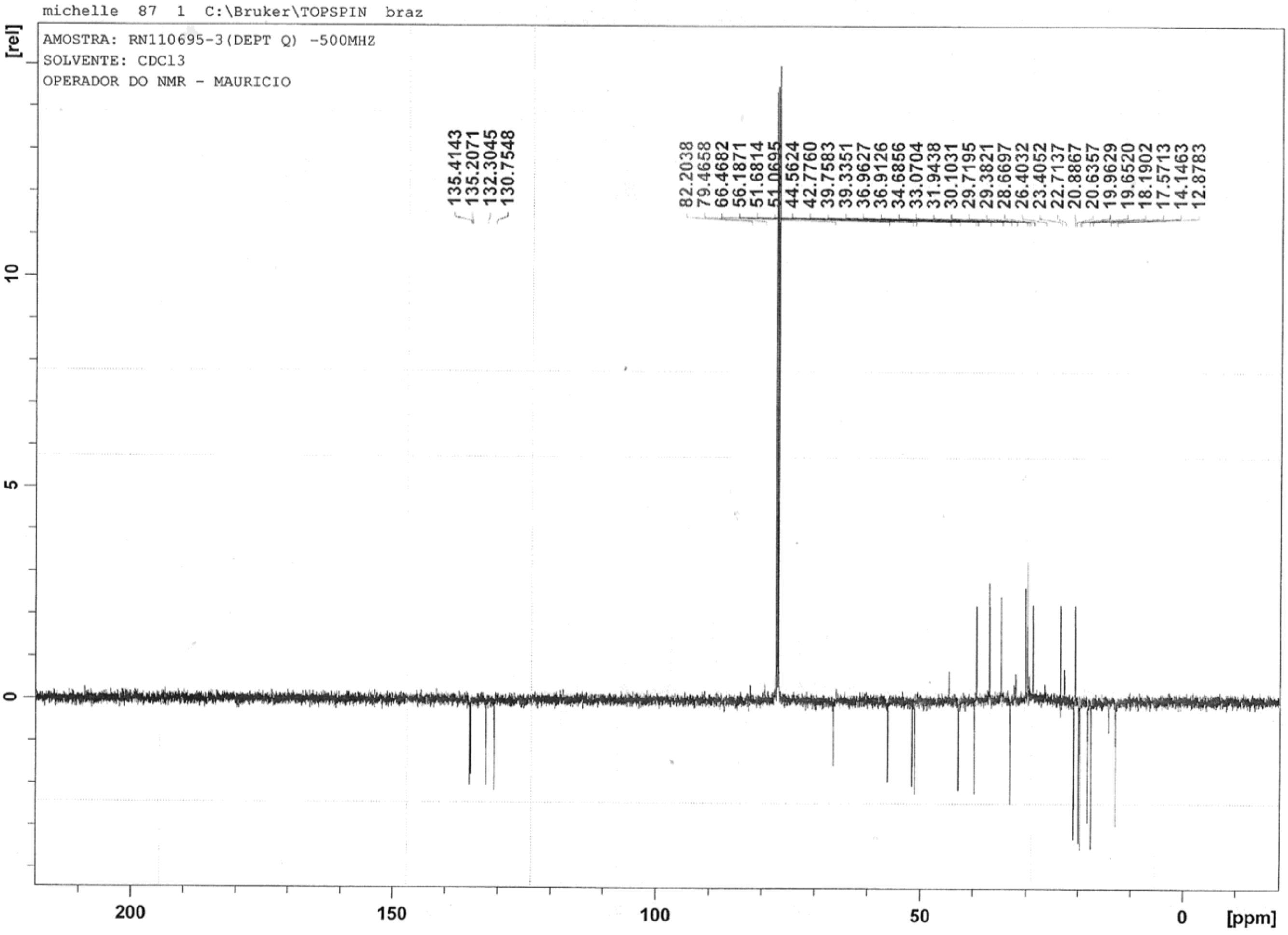

Figure S5. 1H NMR (500 MHz, CDCl3) spectrum of compound **6**.

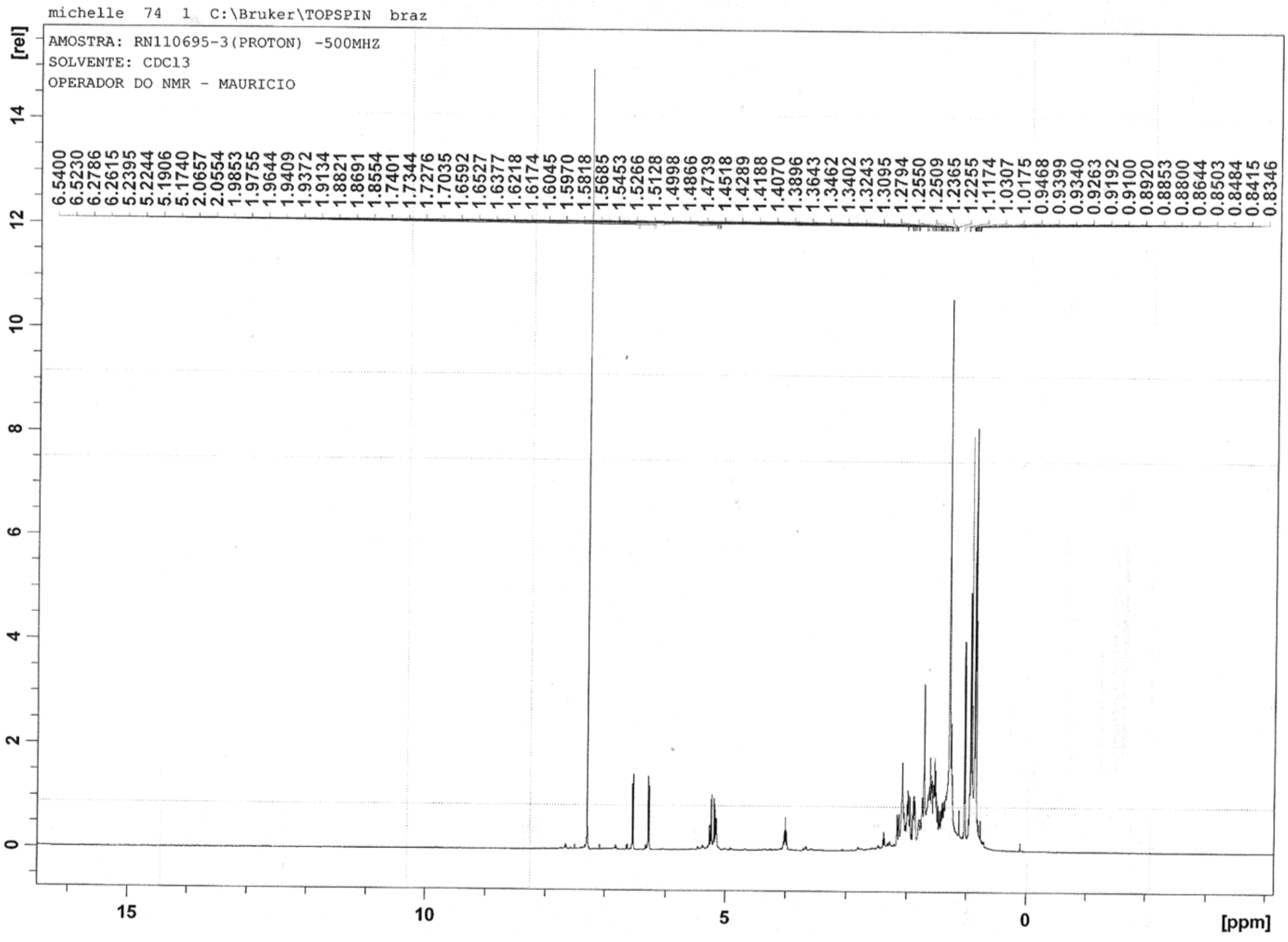

Figure S6. ^1H-^1H COSY NMR (500 MHz, $CDCl_3$) spectrum of compound **6**.

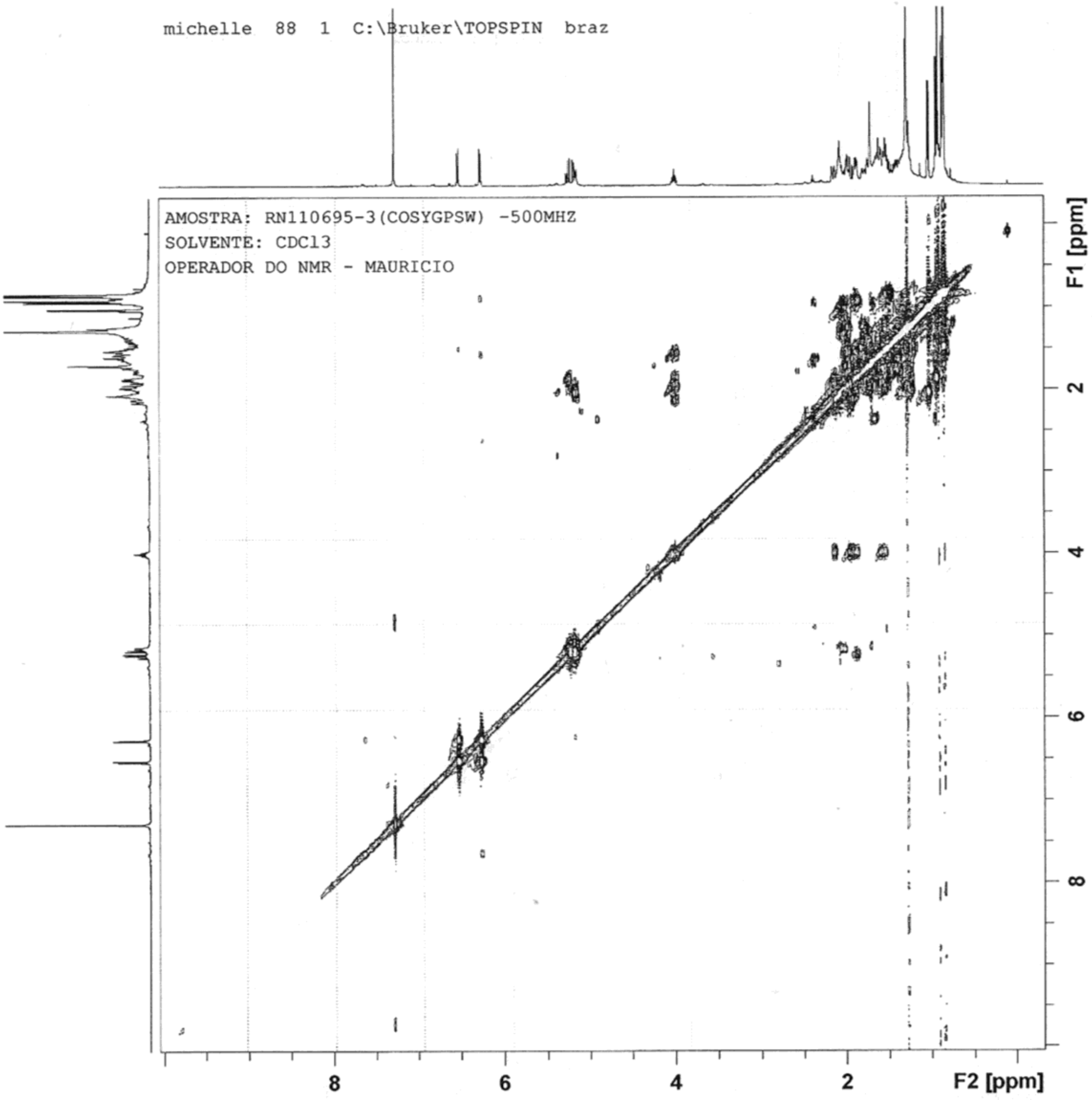

Figure S7. ^{1}H-^{1}H NOESY NMR (500 MHz, $CDCl_3$) spectrum of compound **6**.

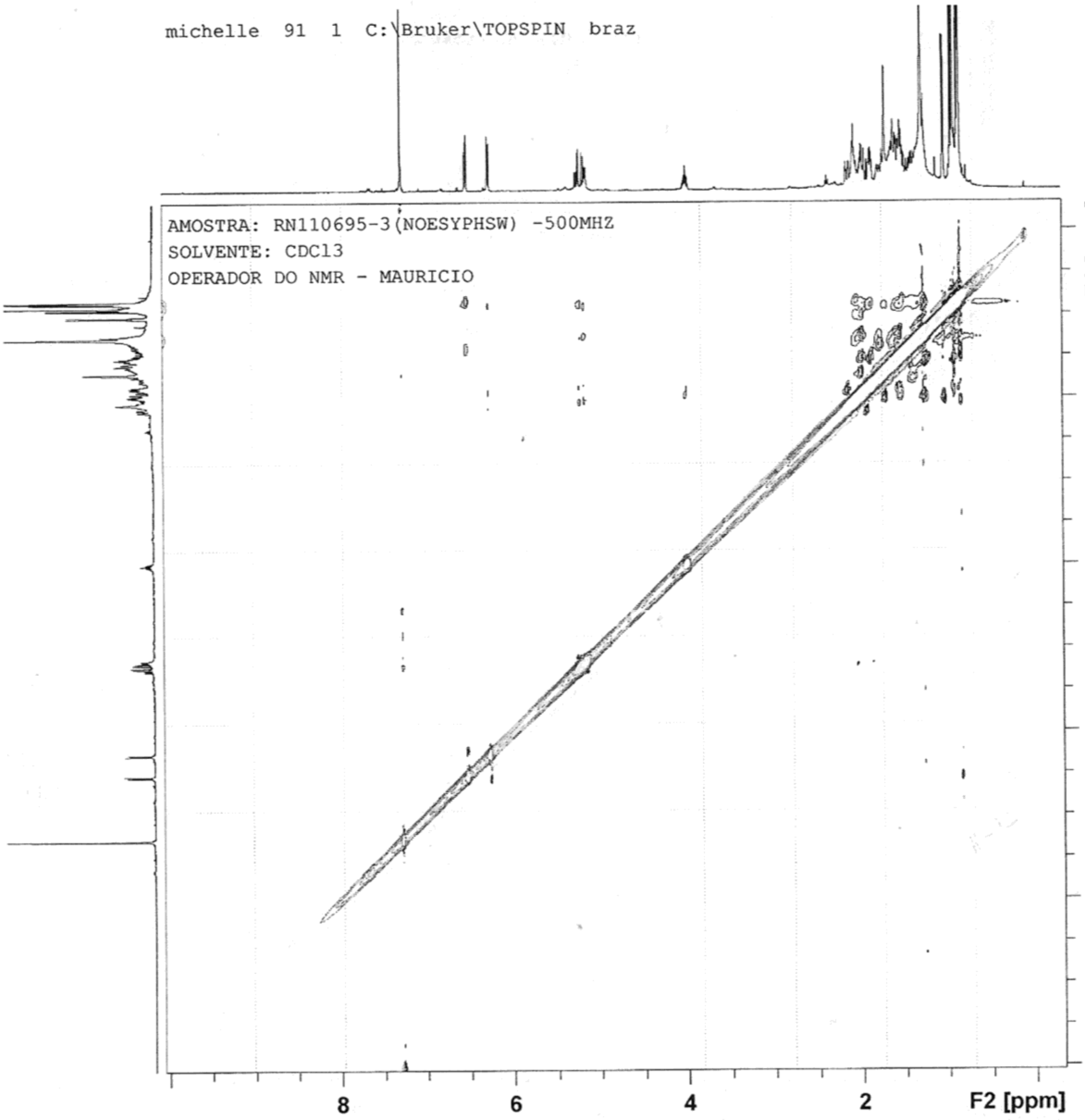

Figure S8. ^{1}H-^{1}H NOESY NMR (500 MHz, $CDCl_3$) spectrum of compound **6**.

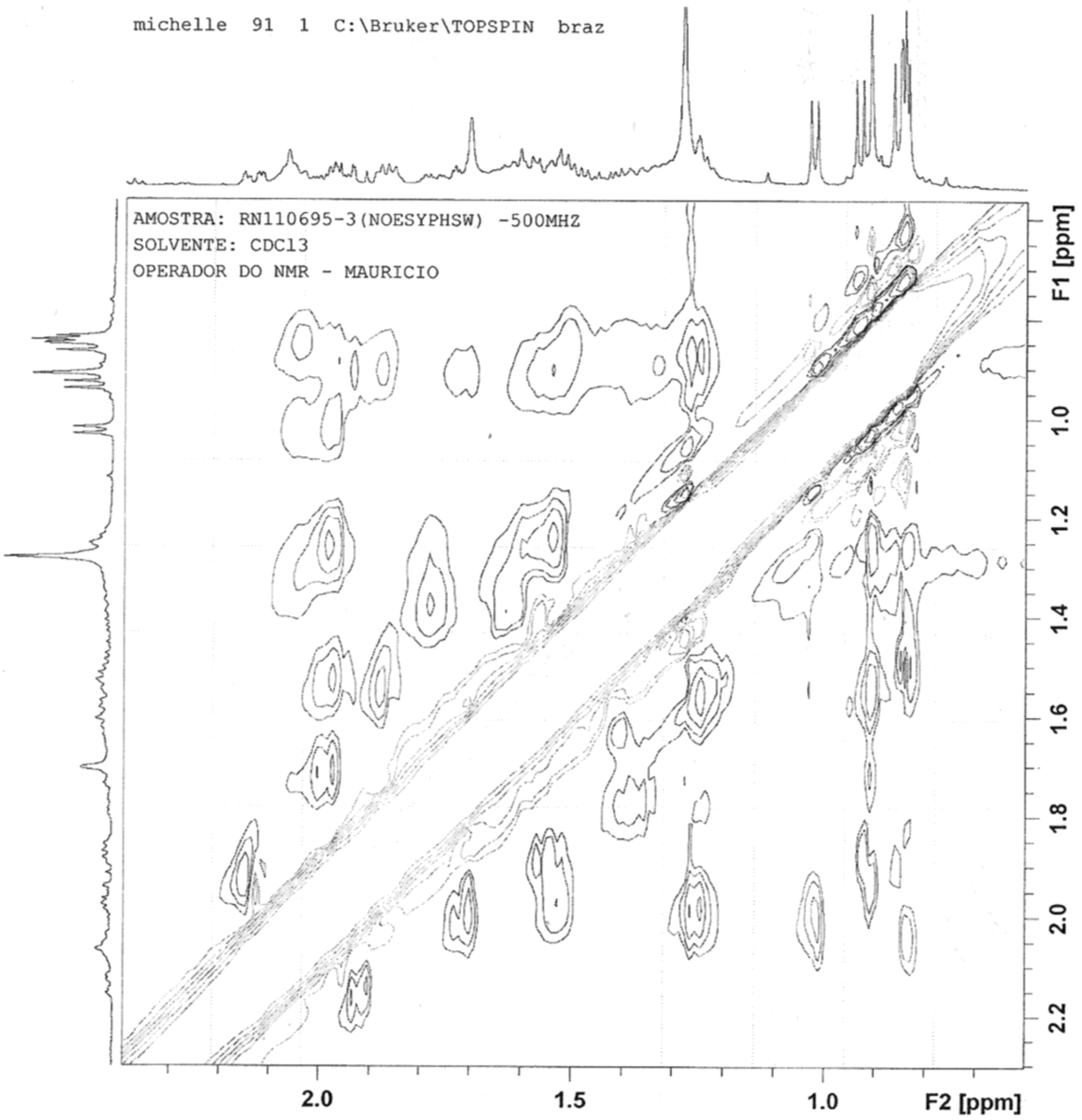

Figure S9. HSQC NMR (500 MHz, $CDCl_3$) spectrum of compound **6**.

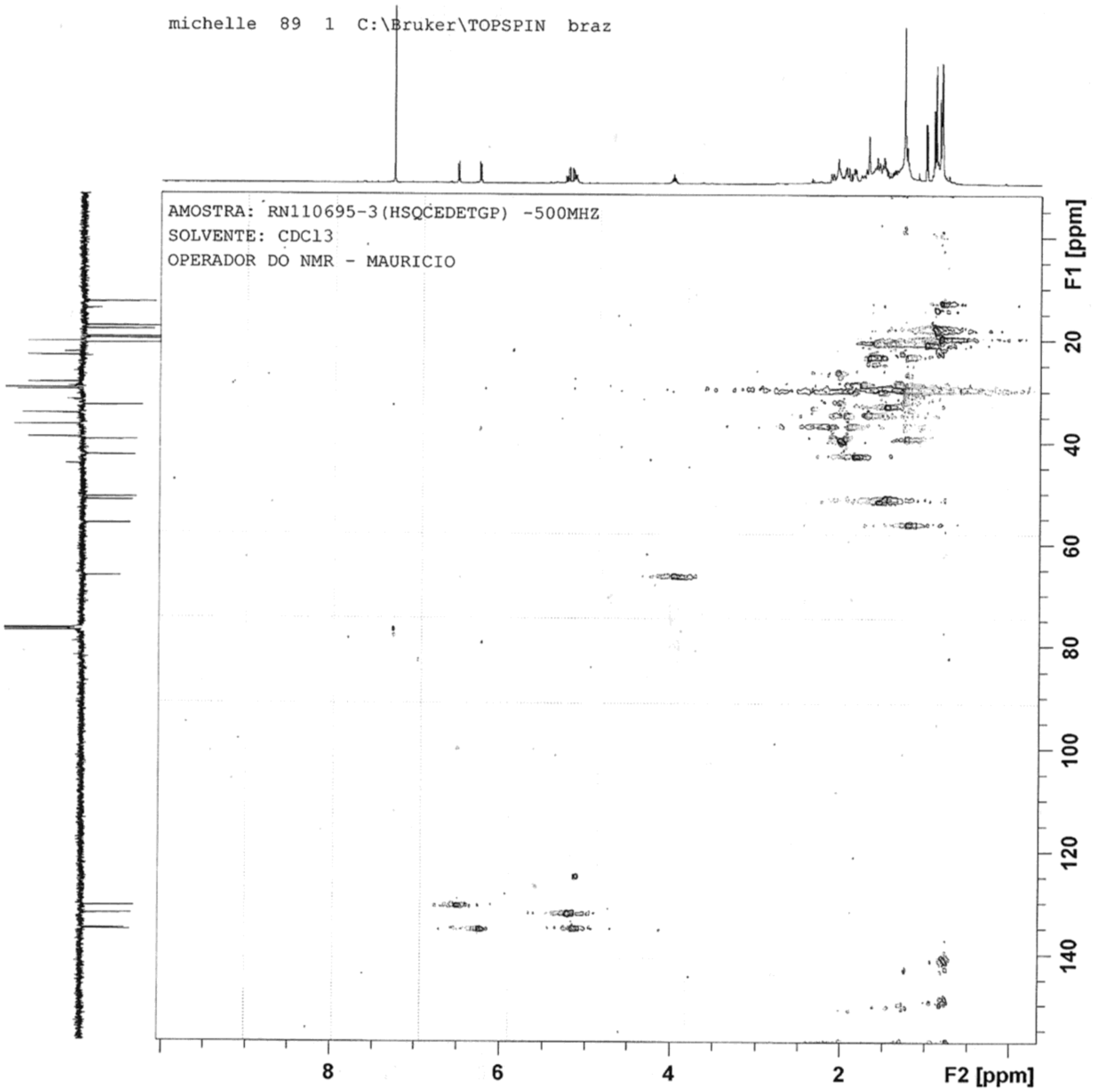

Figure S10. HMBC NMR (500 MHz, $CDCl_3$) spectrum of compound **6**.

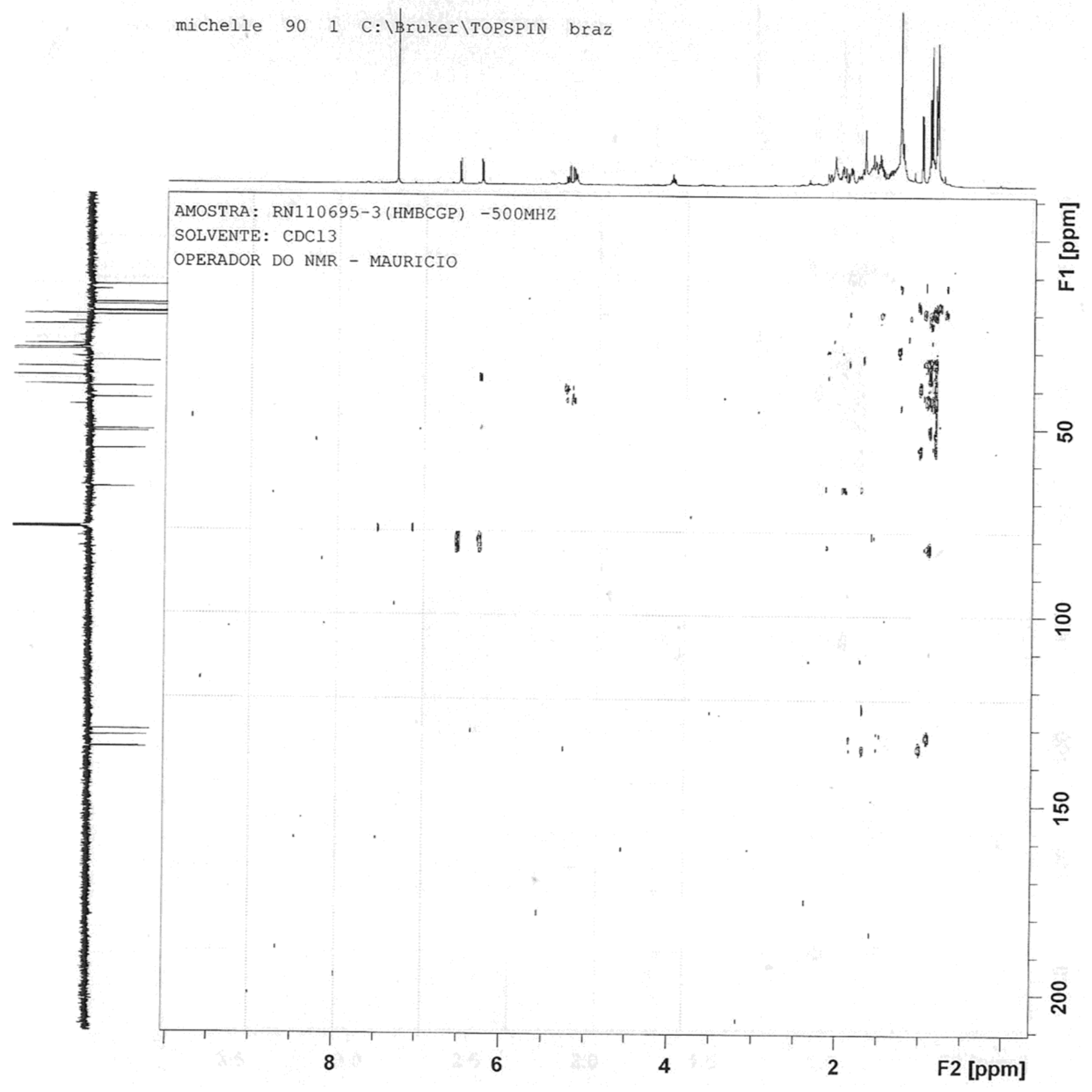

Supplement: Supplementary File 1 [file molecules-19-14637-s001.pdf]
